# Antioxidant and Antimicrobial Activities of *Erodium arborescens* Aerial Part Extracts and Characterization by LC-HESI-MS^2^ of Its Acetone Extract

**DOI:** 10.3390/molecules27144399

**Published:** 2022-07-08

**Authors:** Sonda Samet, Amani Ayachi, Mariam Fourati, Lotfi Mallouli, Noureddine Allouche, Michel Treilhou, Nathan Téné, Raoudha Mezghani-Jarraya

**Affiliations:** 1 Laboratory of Organic Chemistry LR17ES08, Natural Substances Team, Faculty of Sciences of Sfax, University of Sfax, P.B. 1171, Sfax 3000, Tunisia; samet.sonda95@gmail.com (S.S.); amaniayachi21@gmail.com (A.A.); noureddineallouche@yahoo.fr (N.A.); raoudhajarraya@yahoo.fr (R.M.-J.); 2 Laboratory of Microbial Biotechnology and Enzyme Engineering of the Center of Biotechnology of Sfax, University of Sfax-Tunisia, Road of Sidi Mansour Km 6, P.B. 1177, Sfax 3018, Tunisia; mariamfourati@ymail.com (M.F.); lotfi.mallouli@cbs.mrt.tn (L.M.); 3 Equipe BTSB-EA 7417, Institut National Universitaire Jean-François Champollion, Université de Toulouse, Place de Verdun, 81012 Albi, France; michel.treilhou@univ-jfc.fr

**Keywords:** *Erodium arborescens*, acetone extract, LC-HESI-MS^2^, antioxidant activity, antimicrobial effect

## Abstract

The phytochemical analysis of antioxidant and antibacterial activities of *Erodium arborescens* aerial part extracts constitute the focus of this research. The chemical composition of an acetone extract was investigated using LC-HESI-MS^2^, which revealed the presence of 70 compounds. The major identified components were tannin derivatives. Total polyphenol and total flavonoid contents were assessed in plant extracts (hexane, ethyl acetate, acetone and methanol). The results showed that the acetone extract exhibited the highest contents of polyphenols and flavonoids, 895.54 and 36.39 mg QE/g DE, respectively. Furthermore, when compared to other extracts, *Erodium arborescens* acetone extract was endowed with the highest antioxidant activity with 2,2-diphenyl-1-picrylhydrazyl (DPPH), ferric reducing antioxidant power (FRAP) and total antioxidant capacity (TAC) tests. In addition, the four extracts of *Erodium arborescens* showed variable degrees of antimicrobial activity against the tested strains, and the interesting activity was obtained with acetone and methanol extracts.

## 1. Introduction

Phytochemicals from plants are receiving ever a great investigation, which integrates chemistry, botany and the study of secondary metabolites. Secondary metabolites differ from primary metabolites in that they have a wide structural variety, a narrow taxonomic distribution and a narrow range of biological activity [1]. Polyphenols are important plant secondary metabolites with an extensive range of structural characteristics. These are required for various functions in plants, and are responsible for many of the organoleptic and nutritional aspects of plant-derived foods which have numerous applications [2]. Species constituting the Geraniaceae family are rich in polyphenolic compounds, including flavonoids and tannins and are involved in folk medicine as remedies with a large spectrum of indications [3]. This cultural heritage was passed down the centuries long before contemporary medical techniques and medication development strategies were formed. The first step toward understanding the preventive and therapeutic possibilities of using medicinal plants to treat diseases is to preserve this knowledge [4].

*Erodium* is a well-known genus belonging to the Geraniaceae family, and includes more than 70 species which can be found in many continents. The Mediterranean region is considered as the location of many *Erodium* species (more than 60 species). The species can be either annual or perennial and can reach from 2 to 40 cm high [5]. Their leaves are alternate or opposite, pinnate or pinnately lobed, sometimes undivided and the flowers are actinomorphic or slightly zygomorphic; sepals 5, imbricate; petals 5, alternating with glands; fertile stamens 5, antisepalous; staminodes 5, antipetalous; ovary of 5 carpels adnate to central column of flower [6]. Studies on the phytochemical profile of *Erodium* species have shown the presence of tannins and it was reported that ellagic acid was the main phenolic compound present in *Erodium petraeum*, *Erodium botrys*, and *Erodium gruinum.* However, brevifolin was the predominant compound in *Erodium cicutarium* and *Erodium manescavi* [7]. In addition, *Erodium* species showed interesting antioxidant activity and antimicrobial effect against pathogenic microorganisms [7].

To our knowledge, there is no previous works dealing with chemical and biological investigations of *Erodium arborescens*.

This work was designed to investigate, for the first time, the total phenolic and total flavonoid contents as well as the antioxidant and antimicrobial capabilities of extracts obtained from the aerial part of *Erodium arborescens*. The scavenging capacity of hexane, ethyl acetate, acetone and methanol extracts was examined using 2,2-diphenyl-1-picrylhydrazyl (DPPH) free radical scavenging potential, ferric reducing antioxidant power (FRAP) and total antioxidant capacity (CAT) tests. Moreover, the antimicrobial activity was evaluated using the diffusion and micro dilution methods. Finally, the chemical composition of acetone extract was examined using liquid chromatography coupled with linear ion trap mass spectrometry.

## 2. Results and Discussions

### 2.1. Extraction

The extraction yield of phenolic compounds was calculated for each solvent (hexane, ethyl acetate, acetone and methanol) and are shown in Table 1.

We found that the maximum extract yield was obtained with methanol extract while the lowest one was obtained with ethyl acetate extract. The variations between extract yields of the studied plant material could be attributed to the polarity similarity between the solvent and extractable material of plants [8].

### 2.2. Chemical Composition

Polyphenols are a class of natural chemicals that has recently gained a lot of scientific and medicinal attention [9]. The total phenolic contents (TPC) and the total flavonoid contents (TFC) were evaluated in the different extracts.

Table 2 shows that the phenolic contents in acetone extract was significantly the highest one (895.54 mg GAE/g DE) followed by in MeOH (382.42 mg GAE/g DE) then EtOAc (266.20 mg GAE/g DE) extracts; whereas the hexane extract had significantly the lowest phenolic content (205.92 mg GAE/g DE).

The results showed that the TFC varied considerably from 13.03 to 36.39mg in terms of quercetin equivalents/g of dried extract. As was expected for TFC, the acetone extract presented the highest content with 36.39 mg QE/g DE.

### 2.3. Antioxidant Activity

Generally, the antioxidant activity of plant extracts cannot be evaluated using a single approach method due to divergence of phytochemicals, so it is critical to use widely validated assays to evaluate the antioxidant activity. To explain how antioxidants work, different assays such as 2,2-diphenyl-1-picrylhydrazyl test (DPPH), reducing power (FRAP) and total antioxidant capacity (TAC) are the most acknowledged ones and they were consequently utilized in this study to evaluate the antioxidant power of *Erodium arborescens* extracts [10].

#### 2.3.1. Radical-Scavenging Activity (DPPH) Assay

The DPPH test, measured by the molar ratio of antioxidant to DPPH radical required for 50% reduction in DPPH radical concentration, is a reliable method that is widely used. Ascorbic acid was chosen as the antioxidant reference for this test. The degree of discoloration indicates the extract’s scavenging potential which can be quantitatively measured from the changes in absorbance [7,8].

In the present study, the IC_50_ value of each extract was calculated and presented in Table 3. Among all *Erodium arborescens* extracts, the acetone and MeOH ones displayed the most effective (*p* < 0.05) DPPH radical scavenging activity (0.03 mg·mL^−1^) comparable to the positive control (Vitamin C) followed by EtOAc extract (0.039 mg·mL^−1^). Their AAI values ranging between 1 and 2 indicated that they have a strong antioxidant activity. By contrast, the IC_50_ value related to the hexane extract (0.109 mg·mL^−1^) indicated its weak antioxidant activity.

#### 2.3.2. Ferric Reducing Antioxidant Power (FRAP)

The ability of *Erodium arborescens* extracts to reduce ferric ions was determined by using the FRAP assay which evaluates the ability of the tested substances to reduce Fe^3+^ to Fe^2+^. Since the antioxidant property of a substance is based on its reducing capacity, the FRAP assay may therefore serve as a significant indicator of its antioxidant activity. The rapid evaluation of the total antioxidant capacity of plant extracts is frequently tested by this method [11].

**Figure 1:**
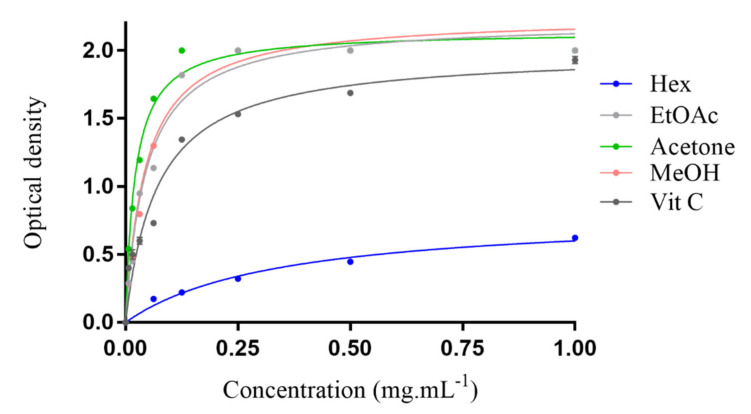
Ferric reducing antioxidant power (FRAP) of *Erodium arborescens* aerial part extracts and vitamin C.

The ferric reducing antioxidant power of all tested extracts is shown in Figure 1. For all extracts, the reducing power increases with increasing concentration. The results clearly indicated that the MeOH, acetone and EtOAc extracts’ reducing powers were much higher than for Vitamin C. The lowest reducing property was observed for the hexane extract.

The following findings confirm that the MeOH and acetone extracts possessed the strongest antioxidant activities.

#### 2.3.3. Total Antioxidant Activity

Total antioxidant capacity (TAC) evaluates the cumulative activity of all antioxidants present in the plant extracts, providing an integrated parameter rather than a simple sum of measurable antioxidants [12]. The total antioxidant capacities of *Erodium arborescens* extracts shown in Table 4 are expressed as mg of gallic acid equivalents per g of dried extract (mg GAE/g DE).

**Figure 2:**
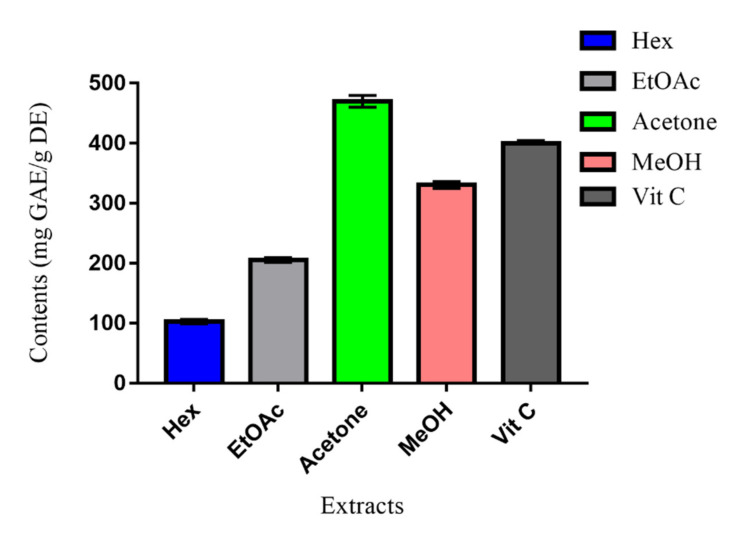
Total antioxidant capacity (TAC) of *Erodium arborescens* aerial part extracts and Vit C.

The results, summarized in Figure 2, indicated higher significant TAC of the MeOH and acetone extracts, respectively, at concentrations of 330 mg GAE/g DE and 470 mg GAE/g DE. Compared to vitamin C (400 mg GAE/g DE), the acetone extract presented the sharpest value. The antioxidant effect of these two extracts may be assigned to their phenolic and flavonoid contents. The order of antioxidant capacities of *Erodium arborescens* extracts was Hex < EtOAc < MeOH < Vit C < Acetone. This order is in good conformity with that of TPC, pointing to the fact that phenolic compounds may be the main antioxidant agents [13].

### 2.4. Correlations between TFC, TPC and Antioxidant Activity

To explore the influence of phytochemical constituents on antioxidant capacity, the correlations between the phenolic contents and antioxidant activity of extracts of the aerial parts of *Erodium arborescens* were determined.

The results in Table 5 revealed important correlations for all the extracts between the TPC and DPPH, and the TFC and DPPH, with correlation coefficients R^2^ = 0.549 and R^2^ = 0.933, respectively. Similarly, an important linear correlation was established between the different phenolic contents of extracts and FRAP; R^2^ = 0.505 for TPC and R^2^ = 0.915 for TFC. For TAC and the different phenol contents, we noticed strong linear correlations with respective coefficients R^2^ = 0.922 (TAC-TPC) and R^2^ = 0.945 (TAC-TFC).

The statistical results obtained in this study indicated the presence of a significant correlation (*p* < 0.05) between FRAP and TAC (R^2^ =0.756). The DPPH test showed a good correlation with FRAP and TAC (R^2^ = 0.994 and R^2^ = 0.798, respectively).

### 2.5. Antimicrobial Activity

The four extracts (hexane, ethyl acetate, acetone and methanol) of *Erodium arborescens* were tested for their antimicrobial activity against six pathogenic microorganisms: two Gram-positive bacteria *Listeria monocytogenes* and *Staphylococcus aureus*; three Gram-negative bacteria *Pseudomonas aeruginosa*, *Salmonella enterica typhimurium* and *Escherichia coli*; and one fungus *Candida albicans*.

*Listeria monocytogenes* causes invasive diseases in humans, especially central nervous system infections [14]. *Staphylococcus aureus* is both a commensal and an extremely versatile pathogen in humans, causing several syndromes such as superficial lesions, deep-seated and systemic infections and toxemic syndromes [15]. *Pseudomonas aeruginosa* is an opportunistic pathogen affecting immunocompromised patients and it is known as the leading cause of morbidity and mortality in cystic fibrosis patients [16]. *Salmonella enterica-typhimurium*, the causative agent of typhoid fever, is a major public health threat and an estimated 27 million cases result in approximately 217,000 deaths annually worldwide due to this pathogenic bacterium [17]. In addition to being an important member of the normal intestinal microflora of humans and other mammals, *E. coli* contains many pathotypes that cause a variety of diseases [18]. *Candida albicans*, the most common human fungal pathogen, is an opportunistic pathogen that can threatenimmunologically weak and immunocompromised people and causes severe, life-threatening bloodstream infections in vulnerable patients [19].

The emergence of these pathogenic microorganisms has led to renewed interest in exploring the potential of bioactive plant compounds for human medicine and food preservation.

As shown in Table 6, the four extracts of *Erodium arborescens* showed variable degrees of antimicrobial activity against the six tested microbial strains.

The hexane extract presented moderate inhibitory activity against the Gram-positive pathogen *L. monocytogenes* and the three Gram-negative pathogenic bacteria *P. aeruginosa*, *S. enterica-typhimurium* and *E. coli*. Ethyl acetate and acetone extracts acted strongly against all tested bacteria and the fungus *C. albicans*. The effects of these two extracts were more pronounced against Gram-negative bacteria than against Gram-positive ones. The strongest activity was obtained against the Gram-negative pathogenic bacterium *P. aeruginosa* with an inhibitory zone of 28 and 30 mm for ethyl acetate and acetone extracts, respectively. Comparing the two, the acetone extract appeared to be more promising than the ethyl acetate extract. The methanol extract exhibited an important inhibitory activity against only pathogenic Gram-negative bacteria and the fungus *C. albicans*. For this extract, the highest activity was observed against *P. aeruginosa*, *S. enterica-typhimurium* and the fungus *C. albicans* with inhibitory zones of 26, 28 and 26 mm, respectively.

Pathogenic Gram-negative bacteria are a real threat to human health and a burden on the health and food industries. Gram-negative bacteria have a thin peptidoglycan layer and have an outer lipid membrane (OM) whereas Gram-positive bacteria have a thick peptidoglycan layer and no outer lipid membrane. The OM of Gram-negative bacteria is an asymmetric bilayer with an inner leaflet of phospholipids and an outer leaflet of lipopolysaccharide [20], which is selectively permeable and thus regulates access to the underlying structures [21]. Moreover, the OM contains proteins called the outer membrane proteins (OMPs) which allow the passage of small molecules such as amino acids and small saccharides. This outer membrane of Gram-negative bacteria constitutes a real and robust permeability barrier that prevents many antibiotics from reaching their intracellular targets [22]. To explain the inhibitory activity of the *Erodium arborescens* methanol extract against Gram-negative bacteria only, and not against Gram-positive ones, one hypothesis is to consider that bioactive constituents of the methanol extract act at the OM level and thus disturb its selectivity function.

Minimum inhibitory concentrations (MICs) of the four extracts (hexane, ethyl acetate, acetone and methanol) of *Erodium arborescens* were assessed by micro-dilution method against four indicator microorganisms: two Gram-positive bacteria *L. monocytogenes* and *S. aureus*, one Gram-negative bacterium *S. enterica-typhimurium;* and a fungus *C. albicans*. As shown in Table 7, the MICs values range from 3.9 to 62.5 µg·mL^−1^ against *L. monocytogenes*, and the lowest MIC value (3.9 µg·mL^−1^) was obtained with acetone extract, which was equal to that of ampicillin and three times lower than that of kanamycin (12.5 µg·mL^−1^). Against *S. aureus*, MICs values were 15.6 and 6.25 µg·mL^−1^ for ethyl acetate and acetone extracts, respectively, and the MIC value of the acetone extract was equal to that of kanamycin. The MICs values range from 3.9 to 62.5 µg·mL^−1^ against the Gram-negative bacterium *S. enterica-typhimurium*, and the lowest MIC was that of the methanol extract (3.9 µg·mL^−1^) equal to the MIC of ampicillin and three times lower than that of kanamycin (12.5 µg·mL^−1^). The MIC of the acetone extract against this Gram-negative bacterium was 6.25 µg·mL^−1^ and was two times lower than that of kanamycin. Concerning MICs against *C. albicans*, values ranged from 1.25 and 6.25 µg·mL^−1^. The lowest MIC was that of methanol extract (1.25 µg·mL^−1^), which was equal to the MIC of the standard fluconazole. The MIC of the acetone extract was 2.5 µg·mL^−1^, which was two times higher than that of fluconazole and five times lower than MIC of ethyl acetate extract (6.25 µg·mL^−1^).

According to previous research, it was reported that the antimicrobial properties of plants have been established to be due, mainly, to geraniin [23] with an MIC of 0.16 mg·mL^−1^ against *C. albicans*. Adding to that, corilagin also accounts for its antimicrobial properties validating its use in traditional medicine for treating infections [24]. In fact, the MICs of corilagin were 31.25 µg·mL^−1^ and 62.5 µg·mL^−1^ against *S. aureus* and *C. albicans.* Although both geraniin and corilagin are major in the acetone extract, the two together proved to be more active with MICs varying from 6.25 to 2.5 µg·mL^−1^ against testing strains. This can be explained by the additive and synergistic effects of each extract constituents to be more effective than the isolated constituents [13].

### 2.6. Identification of Polyphenols in Acetone Extract

**Figure 3:**
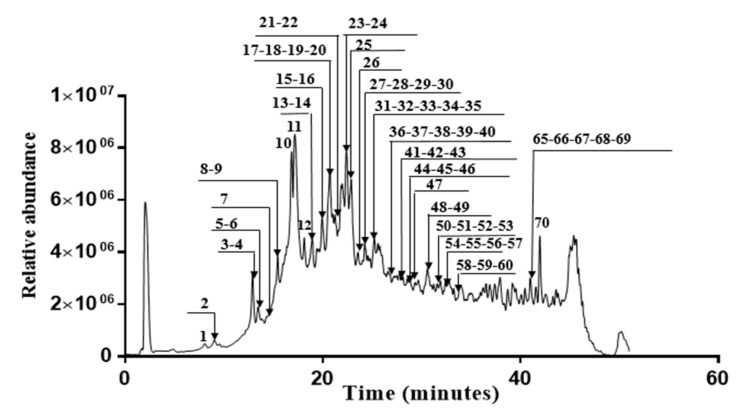
Chromatogram of acetone extract from the aerial parts of *Erodium arborescens* obtained by LC-HESI-MS analysis into negative mode.

In this investigation, fifty polyphenols were identified in the *Erodium arborescens* acetone extract (Figure 3). Hydrolysable tannins, such as simple gallate esters, ellagic acid derivatives and glycosides and different ellagitannins were the main compounds in the acetone extract. HPLC coupled with hot electrospray ionization mass spectrometry was used to characterize all identified compounds listed in Table 8. For each peak, we have indicated the retention time, the relative intensity, the deprotonated mass and the fragment ions generated by MS^2^ operation.

#### 2.6.1. Characterization of Ellagitannins

Gallic acid and its derivatives are often found in *Erodiums* in ester form as hydrolysable tannins (HTs) [25]. HTs can be divided into three groups: galloyl glucoses (GGs) that are galloyl esters of glucose; gallotannins (GTs) that are galloyl glucoses where additional galloyl groups are attached with depside bonds; and ellagitannins (ETs) where two galloyl groups are attached to form hexahydroxydiphenoyl (HHDP) or further oxidized groups such as dehydrohexahydroxydiphenoyl (DHHDP) group [1].

- The precursor ion at *m*/*z* 495 of peak **4** detected at T_R_ = 12.88 min was consistent with the presence of 3,5-di-*O*-galloylquinic acid [26]. The fragmentation of this precursor ion produced ions at *m*/*z* 343 due to the loss of galloyl moiety and at *m*/*z* 325 due to the elimination of a gallic acid molecule.

Geraniin is one of the most abundant compounds that was first isolated from this genus [27]. In this study, geraniin is the main compound in the acetone extract and co-occurs with an isomer named isogeraniin. In addition, several other ETs were identified from the aerial part such as corilagin, geraniinic acids and ascorgeraniin.

- Peak **1** gave the deprotonated molecule [M-H]^−^/*z* 169 at T_R_ = 8.08 min. The major and only fragment was *m*/*z* 125 [M-CO_2_-H]^−^ corresponding to the loss of a carbon dioxide moiety [28].
- Peak **11** mass spectrum shows an ion at *m*/*z* 951 [M-H]^−^ at T_R_ = 17.20 min and, on the basis of the fragmentation pattern and literature data, it was identified as Geraniin [29]. Its MS^2^ spectrum yielded fragments at *m*/*z* 933 due to dehydration [M-H-18], at *m*/*z* 479 due to a loss of HHDP-galloyl moiety and finally at *m*/*z* 301 corresponding to ellagic acid.

The hydrolysis products of geraniin are stable especially corilagin. Other compounds can be produced from geraniin such as brevifolin carboxylic acid. The literature about the phytochemical composition of *Erodium* genus gave an approach to characterize this class of secondary metabolites in *Erodium arborescens*.

- Corilagin at peak **10** has been already well characterized in previous studies [27]. This compound is a complex ellagitannin containing glucose, gallic acid and HHDP. Corilagin was detected in the TIC chromatogram at T_R_ = 16.84 min. Its precursor [M-H]^−^ was *m*/*z* 633 which gave two main fragments at *m*/*z* 463 and 301 due to consecutive losses of gallic acid and galloyl, supporting the identity of this compound.
- Peak **9** had the precursor ion *m*/*z* 247 at T_R_ = 15.51 min and has been tentatively proposed as brevifolin carboxylate [30]. The product ion at *m*/*z* 219 may result from the cleavage of the ester bond and subsequent loss of the CO group from brevifolin carboxylate.
- Another peak **16** was proposed as methyl brevifolin carboxylate [27] with a molecular ion *m*/*z* 305 at T_R_ = 19.93 min. Its MS^2^ spectrum showed a fragment ion at *m*/*z* 273, suggesting the loss of CH_3_OH.
- Peak **19** was observed with *m*/*z* 953 [M-H]^−^ at T_R_ = 20.36 min and the following fragmentations were: *m*/*z* 935 (dehydration), *m*/*z* 633 (loss of HHDP group), *m*/*z* 463 (combined losses of HHDP and galloyl groups) and *m*/*z* 301 (HHDP residue). It can be ascribed, however, to geraniinic acid A [31].
- Peak **26**, generating a molecular ion [M-H]^−^ at *m*/*z* 983, was eluted at T_R_ = 23.80 min and gave one fragment at *m*/*z* 633, indicating the presence of a corilagin unit. It also showed fragments at *m*/*z* 939 due to the loss of CO_2_, and at *m*/*z* 769 due to the loss of gallic acid. Based on fragmentation data, this compound was tentatively identified as a corilagin derivative.
- Peak **36** at T_R_ = 26.08 min can be identified as punicatannin A/B [32]. This identification was confirmed due to its pseudo molecular ion *m*/*z* 997. The MS^2^ spectrum of this compound product ions at *m*/*z* 633 and 301 supported the existence of a corilagin unit.

The DHHDP group is very reactive and its further oxidation yields ETs such as phyllanthusiins B and G. Those compounds were also identified in this extract.

- Peak **14** has a molecular ion [M H]^−^ at *m*/*z* 925 at T_R_ = 18.94 min, which is tentatively assigned to phyllanthusiin C [33], whereas the product fragment at *m*/*z* 907 is due to water loss, which produced the fragment at *m*/*z* 605 after the further loss of HDDP. The fragment at *m*/*z* 435 is the result of the removal of a galloyl group. The peak at *m*/*z* 301 shows the ionized HDDP unit.
- Another peak **15** corresponding to phyllanthusiin G [34] with a deprotonated ion [M-H]^−^ at *m*/*z* 969 at T_R_ = 19.37 min was detected. The MS^2^ spectra generated fragments at *m*/*z* 925 due to the loss of CO_2_ and at *m*/*z* 633 which indicated the presence of a corilagin unit. The aglycone fragment at *m*/*z* 301 confirms the presence of an ellagic acid. Typical losses during fragmentation are galloyl (152 amu), HHDP (302 amu), galloyl glucose (332 amu), HHDP glucose (482 amu) and galloyl-HHDP-glucose (634 amu) [35].
- A precursor ion *m*/*z* 987 (peak **39**) at T_R_ = 26.67 min was fragmented to give an intense fragment at *m*/*z* 955 by losing a CH_3_OH unit (−32 amu), and a fragment at *m*/*z* 653 by removing a HHDP unit. After sequential removal of gallic acid (−170 amu), the remaining fragment was HHDP glucose (*m*/*z* 483). Based on these fragmentations, we note that compound **39** has the same base molecule as ellagitannins and it was thus suggested as an ellagitannin derivative.
- Peak **35** at T_R_ = 25.98 min exhibited an ion [M-H]^−^ at *m*/*z* 965. Its MS^2^ spectrum shows produced fragments at *m*/*z* 933 and *m*/*z* 795 due to the loss of water and gallic acid moieties, respectively, and one major fragment at *m*/*z* 301 which is typical for castalagin [36]. Based on its MS^2^ spectrum, this compound was identified as a castalagin derivative.

#### 2.6.2. Characterization of Flavonoids

A total of sixteen flavonoids were identified on the basis of their MS^2^ fragmentations. They were mostly with *O* and *C*-glycosides. Sugar moieties consist of hexoside, deoxyhexoside and pentoside as deduced from the losses of 162 amu, 146 amu and 132 amu, respectively. Moreover, we observed MS fragmentation patterns characteristic of C-glycosides flavonoids, including dehydration and cross ring cleavage of the glucose moiety that produce cross ring cleavage [M-H-120] and another cross ring cleavage [M-H-90].

Flavanones

*Naringenin derivatives*

- Peaks **33** and **55** have mono-charged molecular ion *m*/*z* 271 at T_R_ = 25.74 and 32.76 min, respectively. Their MS^2^ gave fragments at *m*/*z* 151 and *m*/*z* 165 produced through retro Diels-Alder reactions by breaking two C-C bonds of the C-ring, which gave structurally informative ions of A-ring and B-ring. However, it should be noted that the two compounds exhibited significantly different retention times. According to these findings, it may be possible to attribute these two compounds to isomeric forms of naringenin [37].

Flavonols

*Quercetin derivatives*

- Peak **20** exhibited the [M-H]^−^ ion at *m*/*z* 595 at T_R_ = 20.69 min with its MS^2^ fragment at *m*/*z* 343 due to a loss of a pentose (−132 amu) and a part of hexose moiety (−120 amu), and another fragment at *m*/*z* 301 completing the loss of hexose, indicating that this compound is a quercetin diglycoside [30].
- Peak **54** at T_R_ = 32.61 min, obtained with a molecular ion at *m*/*z* 301, corresponds to quercetin aglycone [37]. Its MS^2^ spectrum gave fragments at *m*/*z* 273 and 257, due to consecutive losses of CO and CO_2_, respectively. Fragments at *m*/*z* 179 and *m*/*z* 151 resulted from breaking two C-C of C-ring, retro cyclisation and the loss of CO, respectively.

*Kaempferol derivatives*

- Peak **31** was identified to kaempferol-*O*-glucoside [38]. Its MS^2^ spectrum gave fragments 327 [M-H-120]^−^ and 285 [M-H-162]^−^.
- Peak **46** had a quasi-molecular ion *m*/*z* 489 at T_R_ = 28.89 min giving a fragment at *m*/*z* 285, probably owing to the removal of acetylhexoside group (204 amu). Kaempferol acetylhexoside [39] might be identified with this molecule. Despite the loss of the acetylhexoside group, which was the most predominant fragment, a small fragment at *m*/*z* 327 was also discovered. In agreement with this hypothesis, we can conclude that the *m*/*z* 327 fragment can be produced as a result of the sugar cleavage. Furthermore, the presence of a fragment at *m*/*z* 285 indicates the presence of the aglycone kaempferol.
- Peak **25** gave [M-H]^−^ at *m*/*z* 579 at T_R_ = 22.89 min. In its MS^2^ spectrum, the ion at *m*/*z* 285 [M-H-(162 + 132)]^−^ was the only one observed, suggesting the presence of hexose and pentose moiety. This compound was attributed to kaempferol-*O*-pentosyl-*O*-hexoside [40].

*Isorhamnetin derivatives*

- Peak **68** produced the deprotonated aglycone at *m*/*z* 315 at T_R_ = 41.42 min. The characteristic product ions at *m*/*z* 300, 271, 255 and 227 led to its identification as isorhamnetin aglycone [41].
- Peak **29** produced a [M-H]^−^ at *m*/*z* 623 at T_R_ = 24.29 min. In its MS^2^ spectrum, a predominant fragment at *m*/*z* 315 [M-H-308]^−^ was observed due to the loss of 308 amu (162 + 146) indicating that a hexose and a deoxyhexose are linked at the same position of the isorhamnetin aglycone. Isorhamnetin [42] as aglycone was confirmed by the presence of fragment at *m*/*z* 271.
- Isorhamnetin-*O*-glucuronide [43] was identified (peak **45**) with a molecular ion *m*/*z* 491 at T_R_ = 28.60 min. Its MS^2^ spectrum showed fragments at *m*/*z* 459 and 323, due to successive losses of CH_3_OH and glucuronide moiety, respctively. The loss of a glucuronic acid from the fragment at *m*/*z* 491 resulted in *m*/*z* 315 as the base peak.
- Peak **58** at T_R_ = 33.28 min was proposed to be an isorhamnetin-*O*-pentosyl-hexoside [44] with *m*/*z* 609. Its MS^2^ showed a fragment at *m*/*z* 477 corresponding to the loss of pentoside [M-H-132]^−^ and a fragment at *m*/*z* 315 which is the result of glucoside loss [M-H-132-162]^−^. The fragment at *m*/*z* 301 represents the loss of CH_3_.

Flavones

*Apigenin derivatives*

- According to the fragmentation of [M-H]^−^ ion at *m*/*z* 563 and the retention times 18.83 and 20.28 min, peaks **13** and **18** were identified as 6-*C*-arabinosyl-8-*C*-glucosyl apigenin [45]. Their MS^2^ data showed fragments at *m*/*z* 473 [M-H-90]^−^ and 443 [M-H-120]^−^, indicating the presence of a *C*-hexosyl unit. Another fragment was observed at *m*/*z* 503 [M-H-60]^−^ corresponding to the fragmentation of pentose. The base peak at *m*/*z* 473 [M-H-90]^−^ and the high abundance of the fragment at *m*/*z* 503 [M-H-60]^−^ revealed the presence of a 6-*C*-pentosyl unit.
- Peak **34** at T_R_ = 25.89 min was identified as apigenin-*O*-hexoside [46] showing an [M-H]^−^ ion at *m*/*z* 431. Its MS^2^ spectrum gave a fragment at *m*/*z* 269 typical to apigenin aglycone, after sequential loss of hexose unit.

*Luteolin derivatives*

- Peak **61** had a molecular ion at *m*/*z* 285 with T_R_ = 37.29 min. Its MS^2^ spectrum showed exhibition of neutral losses of CO (*m*/*z* 257) and CO_2_ (*m*/*z* 241) probably owing to the C ring. Another neutral loss concerns the C_2_H_2_O (*m*/*z* 199), in which cleavage occurs mainly on the C ring followed by a new cyclization implying the B ring. Moreover, the presence of a fragment at *m*/*z* 175 results from the losses of C_3_O_2_ then C_2_H_2_O, and supports the C-ring-localized cleavage for the C_2_H_2_O loss from the [M-H]^−^ ion. This pattern of fragmentation is in concordance with that of luteolin [37].
- Peak **28** at T_R_= 24.23 min showing *m*/*z* 593 was identified as luteolin-*O*-rutinoside [47]. Its MS^2^ spectrum produced an ion at *m*/*z* 285, characteristic of the aglycone (luteolin) and due to a loss of hexoside and pentoside units.
- Peak **70** had a molecular ion at *m*/*z* 313 at T_R_ = 41.95 min. Its MS^2^ spectrum gave a major ion at *m*/*z* 298, corresponding to luteolin genine. This compound was suggested as dimethoxyluteolin [48].

*Acacetin derivatives*

- Peak **49** showed [M-H]^−^ at *m*/*z* 283 at T_R_ = 30.60 min which yielded only a predominant fragment at *m*/*z* 239 due to a loss of CH_3_ and owing to the formation of a very stable anion radical structure. This phenolic compound was described previously and attributed to acacetin [41].

#### 2.6.3. Characterization of Other Phenolic Compounds

- Peak **2** exhibited a molecular ion at *m*/*z* 325 and has been tentatively proposed as galloyl shikimic acid [49]. The MS^2^ spectrum of this compound generated a fragment at *m*/*z* 169, corresponding to the loss of shikimate moiety and the appearance of gallic acid.
- Peak **5** at T_R_ = 13.42 min was identified as dihydroxyl glucosyl cyclohexane [50] with a molecular ion at *m*/*z* 293 and MS^2^ fragments at *m*/*z*: 173 [M-H-120]^−^ (fragmentation in position 1–4 of the glycoside), 131 [M-H-C_2_H_2_O^−^] (the rest of sugar moiety) and 113 [M-H-18]^−^ (dehydration), respectively.
- Peak **6** (T_R_ = 13.69 min) assigned to a galloyl ester [51] generated a molecular ion at *m*/*z* 605. Its MS^2^ spectrum revealed a major fragment at *m*/*z* 453 [M-H-152]^−^ (loss of galloyl) and a minor fragment at *m*/*z* 291 [M-H-162]^−^ (loss of hexoside).
- Another peak **16** was proposed as methyl brevifolincarboxylate [27] at *m*/*z* 305. Its MS^2^ spectrum showed a fragment at *m*/*z* 273 suggesting the loss of CH_3_OH.
- Peaks **62** and **64** presented a molecular ion *m*/*z* 329 at T_R_ = 39.54 and 39.83 min, respectively, and their MS^2^ fragment ions were *m*/*z* 311, 293, 229, 211, 171. Comparing with published data, these two compounds were identified as tricin. It is worth noting that these two compounds appeared at two different retention times, which allow us to suggest that they are two isomers of tricin [52].

It was reported that the main metabolites identified in the acetone extract of the aerial parts were tannins, which present more than 50% of the total identified compounds. The most abundant ellagitannin was geraniin and its hydrolyzed product corilagin.

In computational studies, the antioxidant activity of tannins increases when the number of galloyl groups and the molecular weight increase until the insolubility becomes a limiting factor.

Polyphenols get their biological activity from their phenolic hydroxy groups. The moderate acidity of the phenolic hydroxy group (pKa 8–12) is one of its most essential characteristics, as it allows it to easily donate hydrogen and produce negatively charged phenolate ions [53].

**Table 8:** Identification of compounds from *Erodium arborescens* acetone extract by LC-HESI-MS^2^ (negative mode). The relative peak area indicates the contribution of each compound to all identified compounds in the extract, providing a measure of relative abundance.

	Retention Time T_R_(Min)	Area (%)	[M-H]^−^	MS^2^	Structure	Reference
1.	8.08	0.04	169	125	Gallic acid	[28]
2.	9.05	0.34	325	307/281/169(100)/125	Galloyl shikimic acid I	[49]
3.	12.23	0.25	357	169(100)/125	Unknown gallotannin	[54]
4.	12.88	1.51	495	343(100)/325	3,5-di-*O*-galloyl quinic acid	[26]
5.	13.42	1.66	293	131(100)/113/101	Dihydroxyl glucosyl cyclohexane	[50]
6.	13.69	0.37	605	453(100)/435/393/291/273/247	Galloyl ester	[51]
7.	14.55	0.51	631	613(100)/603/461	Unidentified	
8.	15.47	2.19	291	247	Brevifolin carboxylic acid	[31]
9.	15.51	2.181	247	247(100)/219	Brevifolin carboxylate	
10.	16.84	10.28	633	463/301(100)/275	Corilagin	[27]
11.	17.20	15.46	951	933(100)	Geraniin	[27,29]
12.	18.14	1.94	1109	1049(100)/973/935	Ascorgeraniin	[1]
13.	18.83	1.96	563	545/503/473/443(100)/383/353	6-*C*-arabinosyl-8-*C*-glucosyl apigenin	[45]
14.	18.94	2.33	925	907/605/435/301(100)	Phyllanthusiin B/C	[33]
15.	19.37	1.13	969	951/925(100)/895/877/755/633/301	Phyllanthusiin G	[1,34]
16.	19.93	3.10	305	273	Methyl brevifolin carboxylate	[27]
17.	20.07	1.36	739	593	Kaempferol-*O*-hexosyl-dirhamnoside	[40]
18.	20.28	0.62	563	545/503/473/443(100)/383/353	6-*C*-arabinosyl-8-*C*-glucosyl apigenin	[45]
19.	20.36	1.21	953	935(100)/909/801/651/633/463/301	Geraniinic acid	[1,31]
20.	20.69	1.15	595	343/301(100)	Quercetin-*O*-arabinopyranosyl-galactopyranoside	[30]
21.	21.01	2.76	769	623	Isorhamnetin-glycosyl-dirhamnoside	[55]
22.	21.22	1.71	965	933(100)/613/301	Castalagin derivative	[36]
23.	22.37	14.99	991	973/933(100)/907/825/689/519/353/301	Castalagin derivative	
24.	22.70	3.22	907	755/737/633(100)/587/435/291	HDDP-decarboxy-valoneoyl-glucoside	[31]
25.	22.89	5.59	579	285	Kaempferol-*O*-pentosyl-*O*-hexoside	[40]
26.	23.80	0.29	983	965/939(100)/911/769/681/633/493/467/301	Corilagin derivative	
27.	24.03	0.43	963	945/878/811(100)/793/605/435/291	Unidentified	
28.	24.23	1.23	593	285	Luteolin-*O*-rutinoside	[56]
29.	24.29	0.77	623	357/315(100)/300/271	Isorhamnetin-*O*-rutinoside	[57]
30.	24,59	0.44	917	873/721/445/301(100)	Unidentified	
31.	25.23	1.78	447	327/285(100)/255	Kaempferol-*O*-glucoside	[38]
32.	25.59	1.32	951	907/737/649/587/479/435/335/301(100)	Geraniinic acid B/C (HHDP-valoneoyl-glucoside isomer)	[31]
33.	25.74	0.65	271	177/165/151(100)	Naringenin	[37,58]
34.	25.89	0.75	431	269	Apigenin-*O*-hexoside	[46]
35.	25.98	0.36	965	933/795(100)/301	Castalagin derivatives	[36]
36.	26.08	0.16	997	633(100)/363/301	Punicatannin A/B	[32]
37.	26.33	0.38	909	877/739/615/437/301(100)	Unidentified	
38.	26.59	0.32	553	509/401(100)	2,3-dihydro biapigenin methyl ether	[59]
39.	26.67	0.24	987	955(100)/653/483/301	Ellagitannin derivative	
40.	26.84	0.47	923	879/825/621/577/451/407/353/301(100)	Unidentified	
41.	27.48	0.43	521	331(100)/271	Unidentified	
42.	27.75	0.35	965	921/795(100)/493/301	Unidentified	
43.	27.99	0.14	539	377(100)/307/275	Oleuropein	[60]
44.	28.18	0.25	1005	973/915/301(100)	Unidentified	
45.	28.60	0.54	491	459/323/315(100)	Isorhamnetin-*O*-glucuronide	[43]
46.	28.89	0.22	489	327/285 (100)/255	Kaempferol acetyl-hexoside	[39]
47.	29.69	0.67	523	523(100)/361/313//271/169	Unidentified	
48.	30.44	0.16	987	943/685/641/515/301(100)	Unidentified	
49.	30.60	0.66	283	283(100)/239	Acacetin	[41]
50.	31.24	0.07	679	517(100)/355	Polysaccharide: Glc^1^ → ^4^Hex^1^ → ^6^Hex^1^ → ^6^Hex	[61]
51.	31.55	0.26	299	299/284(100)/271/255/240	Rhamnocitrin	[58]
52.	31.73	0.24	965	795/301(100)	Unidentified	
53.	31.92	0.70	565	550/337/193(100)/175	Unidentified	
54.	32.61	0.43	301	301(100)/273/257/179/151	Quercetin dehydrate	[37]
55.	32.76	0.17	271	177/151(100)	Naringenin	[37]
56.	32.85	0.20	473	455/379/269(100)	Unidentified	
57.	32.95	0.12	461	446/315(100)	Dimethyl ellagic acid pentoside	[39]
58.	33.28	0.24	609	477/315(100)/301	Isorhamnetin-*O*-pentosyl-hexoside	[44]
59.	33.80	0.47	605	452(100)/329/271	Unidentified	
60.	33.99	0.21	563	548/518/337/235/193(100)/175	Unidentified	
61.	37.29	0.29	285	257/241(100)/199/189/175	Luteolin	[37]
62.	39.54	0.04	329	311/293/229(100)/211/171	Tricin	[52]
63.	39.69	0.10	301	301/283/257/191/151/137(100)	Ellagic acid	[48]]
64.	39.83	0.15	329	311/293/229(100)/211/171	Tricin	[52]
65.	40.46	0.85	357	357/339/285/151/109	Unidentified	
66.	41.00	0.79	282	No fragmentation	Unidentified	
67.	41.28	0.18	441	371(100)/369	Unidentified	
68.	41.42	0.14	315	315/300(100)/271	Isorhamnetin	[41,48]
69.	41.59	0.69	299	No fragmentation	Unidentified	
70.	41.95	2.42	313	313/298(100)	Dimethoxyluteolin	[48]

## 3. Materials and Methods

### 3.1. Collection of Plant Material

The aerial part (stems and leaves) of *Erodium arborescens* were collected in February 2020 from southeastern Tunisia, Sfax; 34°44′52.249″ N 10°45′58.187″ E. This plant was authenticated by Dr. Zouhair Bouallagi, Department of Biology, Faculty of Sciences of Sfax, and its voucher specimen (LCSN 153) was deposited in the Herbarium of the Laboratory of Organic Chemistry (LC17ES08), Department of Chemistry, Faculty of Sciences of Sfax; University of Sfax, Tunisia.

### 3.2. Extraction

The plant was harvested and then placed in shadow at 25 °C until it was completely dry. Exactly 1 kg of *E. arborescens* aerial part was milled and extracted three times by maceration during 24 h using different solvents of increasing polarity: hexane, ethyl acetate, acetone and methanol. The extraction yields were then determined using Equation (1):

Extraction Yield (%) = (Dry extract weight/dry starting material weight) × 100(1)

### 3.3. Chemical Composition

All assays were carried out in triplicate and the results are expressed as mean values ± standard deviations. Higher absorbance indicates higher reducing power.

#### 3.3.1. Determination of Total Phenolic Contents (TPC)

The Folin–Ciocalteu colorimetric method was used to determine total phenolic contents with slight modifications [62] Gallic acid was used as a standard phenolic compound by dissolving gallic acid (0.2 mg) in ethanol (1 mL) then diluting to prepare different concentrations. Briefly, 50 μL of each extract (1 mg·mL^−1^ in ethanol) were added to 2.5 mL of Folin–Ciocalteu reagent (10 times diluted). After 5 min, 2 mL of saturated sodium carbonate solution (75 g·L^−1^) were added. The mixtures were incubated at 30 °C for 1.5 h and the absorbance of the resulting solution was measured at 765 nm. The amounts of phenolic compounds in plant extracts were expressed as milligrams gallic acid equivalents per gram of dry extract (mg GAE/g DE).

#### 3.3.2. Determination of Total Flavonoid Contents (TF)

Total flavonoid contents were determined by aluminum chloride colorimetric assay based on the formation of a complex flavonoid-aluminum with some modifications [63]. Quercetin solution (1 mg·mL^−1^) and its different concentrations were used to make the calibration curve. An amount of 0.25 mL of each extract was mixed with 75 µL of 5% NaNO_2_ for 6 min. After that, 75 µL of 10% AlCl_3_ was added to react for another 6 min. The reaction was stopped by addition of 500 µL of 4% NaOH and the total volume was topped up to 2.5 mL with 60% ethanol. The absorbance was measured after 15 min at 510 nm. Total flavonoid contents were expressed as milligrams quercetin equivalents per gram dry extract (mg QE/g DE).

### 3.4. Antioxidant Activity

The assays were carried out in triplicate and the results are expressed as mean values ± standard deviations. Higher absorbance indicates higher reducing power.

#### 3.4.1. DPPH Antiradical Activity

This method was adapted as reported by Mhalla et al. [64]. Briefly, 50 μL of various concentrations (0.0625; 0.125; 0.25; 0.5 and 1 mg·mL^−1^) of each extract was added to 2 mL of a DPPH solution (0.04 g·L^−1^ in ethanol) followed by 30 min incubation in the dark at room temperature. Ascorbic acid was used as a positive control. The absorbance was measured at 517 nm using a spectrophotometer against the corresponding blank containing ethanol without DPPH solution. The percentage of inhibition (PI %) of DPPH radicals was calculated using the Equation (2):

(2) $$\mathrm{PI}\left(\%\right)=\frac{A_{\mathrm{Control}\;-\;A_{\mathrm{Sample}}}}{A_{\mathrm{Control}}}\times 100$$

IC_50_ was determined by extrapolation from linear regression analysis as the antioxidant concentration reducing 50% of DPPH free radicals.

The results were expressed as the antioxidant activity index (AAI) using the following Equation (3):

(3) $$\mathrm{AAI}=\frac{\mathrm{Final}\;\mathrm{concentration}\;\mathrm{of}\;\mathrm{DPPH}}{{\mathrm{IC}}_{50}}$$

According to the AAI values, we considered:

AAI < 0.5: poor antioxidant activity
0.5 ≤ AAI ≤ 1: moderate antioxidant activity
1.0 ≤ AAI ≤ 2.0: strong antioxidant activity
AAI > 2.0: very strong antioxidant activity
DPPH and IC_50_ are expressed in µg·mL^−1^.

#### 3.4.2. Reducing Power Assay

The reducing power was determined according to the method of Ferreira et al. [65]. Testing solutions of each extract were prepared by mixing 2.5 mL of its different concentrations with 2.5 mL of 200 mmol·L^−1^ sodium phosphate buffer (pH 6.6) and 2.5 mL of 1% potassium ferricyanide. After incubation at 50 °C for 20 min, 2.5 mL of 10% trichloroacetic acid (*w*/*v*) were added followed by centrifugation at 650 rpm for 10 min. Next, 1 mL deionised water and 0.2 mL of 0.1% of ferric chloride were mixed with 1 mL of the upper layer. The absorbance was measured at 700 nm against a blank prepared with the same solution in which the extract was replaced with distilled water. Ascorbic acid was used as a positive control.

#### 3.4.3. Total Antioxidant Capacity

Total antioxidant capacity was carried out according to Prieto et al. [66,67]. An aliquot of 0.1 mL of sample solution containing each extract was combined with 1 mL of reagent solution (0.6 M sulfuric acid, 28 mM sodium phosphate, and 4 mM ammonium molybdate). The testing solution was incubated in a water bath at 95 °C for 90 min. After cooling at room temperature, the absorbance was measured at 695 nm against a blank containing 1 mL of reagent solution in which the extract had been replaced with the appropriate volume of the same solvent used for the sample. Gallic acid was used as standard in the assay.

### 3.5. Analysis of Individual Phenolic Compounds by Analytical LC–HESI–MS

Thermo Scientific LTQ XL MS was used to explore the composition of the aerial part acetone extract. The LC system was equipped with an electrospray ionization source (ESI). Spectra were recorded in negative ion mode, monitored and processed using Thermo Xcalibur Roadmap software. The VC-P10-A pump system, the VC-A12-A auto sampler and the VC-C10-A column were the main elements of the LC system. For analysis, Surveyor HPLC was provided with a C18 (2 µm, 150 mm × 2.1 mm) reversed phase Acclaim column (ThermoFisher) at 30 °C. The solvents were A (0.1% formic acid in water–ACN, 95–5, *v*/*v*) and B (0.1% formic acid in acetonitrile, *v*/*v*). The elution gradient established was from 0 to 40% of solvent B during 40 min, from 40 to 100% B over 2 min and maintained for 3 min before returning to initial conditions. The flow rate of the mobile phase was 0.2 mL·min^−1^, and the injection volume was 20 µL. High-purity nitrogen served as the nebulizer and auxiliary gas for the HESI source, and the capillary temperature was calibrated at 300 °C. The ion spray voltage was set at 3.5 V. The sheath and auxiliary gas were set at 50 and 5 psi, respectively. The acquisition range was from 50 to 1200 *m*/*z*. The method combined full scans and MS^2^ experiments using a collision energy ranging from 10 to 35 eV, depending on the molecular mass of compounds.

### 3.6. Antimicrobial Activity

#### 3.6.1. Microorganisms, Media and Growth Conditions

Antimicrobial activity was determined against six indicator microorganisms which are: two Gram-positive bacteria *Listeria monocytogenes* (*L. monocytogenes*) ATCC 19117 and *Staphylococcus aureus* (*S. aureus*) ATCC6538; three Gram-negative bacteria *Pseudomonas aeruginosa* (*P. aeruginosa*) ATCC 49189, *Salmonella enterica typhimurium* (*S. enterica-typhimurium*) ATCC 14028 and *Escherichia coli* (*E. coli*) ATCC 8739; and the fungus *Candida albicans* (*C. albicans*) ATCC 10231. All these indicator cells were obtained from International Culture Collections (ATCC) and local culture collection of the Laboratory of Microbial Biotechnology and Enzyme Engineering of the Center of Biotechnology of Sfax-Tunisia.

The microorganisms were grown overnight in Luria Bertani (LB) medium (g·L^−1^: peptone 10; yeast extract 5 and NaCl 5, pH 7.2) under aerobic condition and constant agitation (200 rpm) at 30 °C for *L. monocytogenes* and *S. enterica-typhimurium*, at 37 °C for *E. coli*, *S. aureus* and *P. aeruginosa*. Then, they were diluted 1:100 in LB media and incubated for 5 h under constant agitation at the appropriate temperature. In addition, *C. albicans* was cultured at 30 °C on Sabouraud medium (g·L^−1^: dextrose 40, peptone 10, pH 5.6) under aerobic condition and constant agitation, then diluted 1:50 in the same medium and incubated for 5 h under agitation.

#### 3.6.2. Agar Well Diffusion Method

For the determination of the antimicrobial activity of the extracts, the agar well diffusion method was employed according to Guven et al. (2006) [68]. Briefly, 15 mL of molten agar (45 °C) were poured into sterile Petri dishes (Ø 90 mm). A volume of 50 μL of 5 h-old culture of each tested bacteria and 100 μL of 5 h-old culture of the fungus *C. albicans* were evenly spread onto the surface of the agar plates of LB, using agar medium for bacteria and Sabouraud agar medium for *C. albicans*. Once the plates had been aseptically dried, 5 mm wells were punched into the agar with a sterile cork borer. Each extract was dissolved in dimethyl sulfoxide (DMSO–water, 1–9; *v*/*v*) to a final concentration of 1 mg·mL^−1^, filtered through 0.22 μm pore-size black polycarbonate filters (Millipore, Fontenay sous Bois, France), and finally 100 μL of each obtained extract solution was placed into the corresponding well. After staying at 4 °C for 2 h, the plates were incubated at the appropriate temperature during 24 h for bacterial strains, and during 48 h for *C. albicans*. The antimicrobial activity was assayed by measuring in mm the diameter of the inhibition zone formed around the wells.

#### 3.6.3. Minimum Inhibitory Concentrations (MICs)

MICs of the extract solutions and the standards ampicillin, kanamycin and fluconazole (stock solutions at 20 mg·mL^−1^) were determined against four microorganisms: two Gram-positive bacteria *L. monocytogenes* and *S. aureus*, one Gram-negative bacterium *S. enterica-typhimurium* and one fungus *C. albicans*, following Sellem et al. [69]. The test was performed in sterile 96-well microplates with a final volume in microplate well of 100 μL. Extract solutions and standards were serially diluted with dimethyl sulfoxide (DMSO). To each test well, cell suspension was added to a final inoculum concentration of 106 colony forming unit (CFU)/mL of the studied indicator microorganism. The plates were then incubated at appropriate growth conditions of the corresponding indicator microorganism. The MIC was defined as the lowest concentration of the extract solution and standard at which the microorganism does not demonstrate visible growth after incubation. A volume of 25 μL of thiazolyl blue tetrazolium bromide (MTT) at 0.5 mg.mL^−1^ were added to the wells and incubated at room temperature for 30 min. The colorless tetrazolium salt acts as an electron acceptor and was reduced to a red-colored formazan product by the indicator microorganisms. When microbial growth was inhibited, the solution in the well remained clear after incubation with MTT.

For the antimicrobial activity determination (inhibition zones and CMIs), each experiment was carried out simultaneously three times under the same conditions. The obtained diameters of inhibition zones reported in mm of the three tests were quite similar and the reported results are the average of the three experiments. Concerning MICs values reported in µg·mL^−1^, the three analyses were identical.

## 4. Statistical Analysis

Replicate errors were in all cases < 10% (*n* = 3). The differences were analyzed using Duncan and Tukey’s post hoc tests for multiple comparisons with *p* < 0.05. The Statistical Product and Service Solutions program (SPSS) version 20 was used to analyze the differences and calculate the correlation coefficients R^2^ in order to highlight on the one hand, the correlation between the different phenolic contents of all extracts and their antioxidant activity, and the correlation between the different antioxidant activity tests, on the other.

## 5. Conclusions

This research presents an examination of the polyphenol composition of *Erodium arborescens* aerial part acetone extracts, and demonstrates the identification of fifty one compounds grouped into flavonoids, derivatives of phenolic acids and ellagitannins.

In addition, the study revealed that the antioxidant activity of the acetone and methanol extracts is in agreement with the highest amounts of phenolic and flavonoid contents, which exhibited the greatest antioxidant activity in the scavenging of DPPH free radical, ferric reducing antioxidant power (FRAP) and total antioxidant capacity (TAC) tests.

The obtained results yielded high antimicrobial activity, since most of the microbial strains selected for this study have an effective sensitivity to the tested extracts. Acetone and methanol extracts were demonstrated as inhibitors of *L. monocytogenes*, *S. aureus*, *S. enterica typhimurium* and *C. albicans strains*. The antimicrobial potential of the acetone extract may be associated with its richness of phenolic constituents, including corilagin and geraniin, according to LC/MS-MS analysis.

Finally, future studies should focus on the isolation of active compounds and the investigation of more pharmacological activities.

## Figures and Tables

**Table 1:** Yields (%) of *Erodium arborescens* aerial part extracts.

Extracts	Yields (%)
Hex	0.92
EtOAc	0.59
Acetone	1.77
MeOH	2.66

Hex: hexane. EtOAc: ethyl acetate. Acetone: acetone. MeOH: methanol.

**Table 2:** Total phenolic and total flavonoid contents in *Erodium arborescens* aerial part extracts.

Extracts	TPC (mg GAE/g DE)	TFC (mg QE/g DE)
Hex	205.92 ± 4.20 ^d^	13.03 ± 0.23 ^d^
EtOAc	266.20 ± 4.17 ^c^	26.38 ± 0.51 ^c^
Acetone	895.54 ± 5.01 ^a^	36.39 ± 0.43 ^a^
MeOH	382.42 ± 8.57 ^b^	29.85 ± 0.40 ^b^

TPC: Total phenolic content; TFC: Total flavonoid content; GAE: Gallic acid equivalent; QE: Quercetin equivalent; DE: Dried extract. Values expressed are means ± S.D. (*n* = 3). The differences were analyzed using Duncan and Tukey’s post hoc test for multiple comparisons with *p* < 0.05: ^a^: strong significance, ^b^: high significance, ^c^: modest significance, ^d^: low significance.

**Table 3:** Radical-scavenging activity (DPPH) assay of *Erodium arborescens* aerial part extracts compared to Vitamin C as reference.

Extract/Vit C	IC_50_ (mg·mL^−1^)	AAI
Hex	0.109 ± 0.001 ^c^	0.36
EtOAc	0.039 ± 0.001 ^b^	1.02
Acetone	0.030 ± 0.002 ^a^	1.33
MeOH	0.030 ± 0.001 ^a^	1.33
Vit C	0.029 ± 0.002	1.37

Vit C: reference antioxidant. AAI: antioxidant activity index. Values expressed are means ± S.D (*n* = 3). The differences were analyzed using Duncan and Tukey’s post hoc test for multiple comparisons with *p* < 0.05: ^a^: strong significance, ^b^: high significance, ^c^: low significance.

**Table 4:** Total antioxidant activity of *Erodium arborescens* aerial part extracts and of vitamin C.

Extract	Hex	EtOAc	Acetone	MeOH	Vit C
TAC (mg GAE/g DE)	103.03 ± 1.99 ^d^	205.47 ± 2.31 ^c^	470.09 ± 5.66 ^a^	330.59 ± 3.25 ^b^	400 ± 1.25

Values expressed are means ± S.D. (*n* = 3). The differences were analyzed using Duncan and Tukey’s post hoc test for multiple comparisons with *p* < 0.05: ^a^: strong significance, ^b^: high significance, ^c^: modest significance, ^d^: low significance.

**Table 5:** Correlation among phenolic compounds and assays *.

Extract	TPC	TFC	DPPH	FRAP	TAC
TPC	1	0.805 **	0.549	0.505	0.922 **
TFC		1	0.933 **	0.915 **	0.945 **
DPPH			1	0.994 **	0.798 **
FRAP				1	0.756 **
TAC					1

**: The correlation is significant at the 0.01 level. TPC: Total phenolic content; TFC: Total flavonoids content; *: Data show the Pearson correlation coefficients (R^2^) between the parameters (*p* < 0.05).

**Table 6:** Antimicrobial activity of *Erodium arborescens* aerial part extracts against the six tested indicator microorganisms. The diameters of inhibition zones were reported in millimeter (mm).

Microorganism/ Extract	*L. monocytogenes*	*S. aureus*	*P. aeruginosa*	*S. enterica* *typhimurium*	*E. coli*	*C. albicans*
Hex	12	-	14	12	12	-
EtOAc	20	20	28	22	25	22
Acetone	26	22	30	24	26	24
MeOH	-	-	26	28	20	26

“-” means that the extract did not provide an antimicrobial activity against different strains.

**Table 7:** Minimum Inhibitory Concentrations (MICs), expressed in µg·mL^−1^, of *Erodium arborescens* aerial part extracts compared to three standards (ampicillin, kanamycin and fluconazole) against *L. monocytogenes*, *S. aureus*, *S. enterica-typhimurium* and *C. albicans*.

Extracts and Standards	Microorganisms			
	*L. monocytogenes*	*S. aureus*	*S. enterica-* *typhimurium*	*C. albicans*
Hex	62.50	-	62.50	-
EtOAc	6.25	15.60	6.25	6.25
Acetone	3.90	6.25	6.25	2.50
MeOH	-	-	3.90	1.25
Ampicillin	3.90	1.95	3.90	-
Kanamycin	12.50	6.25	12.50	-
Fluconazole	-	-	-	1.25

“-” means that the extract did not provide any inhibitory concentration against tested strains.

## Data Availability

Not applicable.
